# Pathogenetic and Prognostic Factors for Neonatal Gastric Perforation: Personal Experience and Systematic Review of the Literature

**DOI:** 10.3389/fped.2018.00061

**Published:** 2018-04-04

**Authors:** Chiara Iacusso, Alessandro Boscarelli, Fabio Fusaro, Pietro Bagolan, Francesco Morini

**Affiliations:** Department of Medical and Surgical Neonatology, Bambino Gesù Children’s Hospital, IRCCS, Rome, Italy

**Keywords:** neonate, gastric perforation, prematurity, gastrointestinal emergency, pneumoperitoneum

## Abstract

**Introduction:**

Neonatal gastric perforation (NGP) is a rare entity. Our aim was to report our experience and review the recent literature to characterize NGP, describe associated factors, and define prognostic factors.

**Materials and methods:**

Retrospective review of all consecutive patients with NGP treated between June 2009 and December 2017 in a third level pediatric hospital. In addition, a systematic review of Medline and Scopus database was performed using a defined strategy. All articles referring to NGP published between 2005 and 2017 were retrieved. Variables considered: prematurity (<37 weeks gestation), birth weight (BW), Apgar score, prenatal complications, age at diagnosis, bag ventilation, pathogenetic events, site of perforation, treatment of perforation, sepsis, and outcome. Mann–Whitney or Fisher’s test were used as appropriate. Results are median (range) or prevalence.

**Results:**

Between 2009 and 2016 we treated 8 consecutive patients for NGP and 199 further cases were retrieved from the systematic review (total of 207 patients). Overall survival was 73%. Most frequently reported pathogenesis: iatrogenic (20 patients), hypoxic/ischemic or infection stress (13 patients), duodenal/jejunal obstruction (11 patients), drugs (11 patients), esophageal atresia (10 patients). 60% patients had only primary repair of the perforation as gastric surgery. Sepsis developed in 56 patients (34%).

**Conclusion:**

Although the pathogenesis of NGP is pleomorphic, prematurity and low BWs are frequent in these patients. Reviewing our experience and the available literature, none of the variables considered, but sepsis was associated with mortality.

## Introduction

Neonatal gastric perforation (NGP) is an uncommon life-threatening entity in newborns, potentially challenging for the treating physicians. The reported incidence is 1:5,000 live births and NGP represents 7% of all gastrointestinal perforations in the newborn (1). The first reported case of spontaneous NGP was in 1825 by Siebold (2). Since then, the number of neonatal gastrointestinal perforation (including NGP) is in progressive rise, may be due to the increasing number of very-low birth weight and premature infants, while the mortality related to NGP has dropped by about tenfold, probably due to the improvement of the quality of the neonatal intensive care (3). Despite the increase in prevalence, NGP remains a relatively rare condition, with mainly case reports or small series reported. As a consequence, several aspects of NGP are still unclear, ranging from the etiology to its best treatment.

The aim of this study was to report our own experience with NGP and review the available literature on the issue, in an attempt to better characterize NGP, describe associated factors and treatments proposed, and define prognostic aspects.

## Materials and Methods

A retrospective analysis of all consecutive patients with NGP admitted to our third level pediatric hospital between January 2009 and December 2017 was carried out. The time span was chosen to have available electronic records for all patients. Variables considered included: prematurity (<37 weeks gestation), birth weight (BW), Apgar score at 1′ and 5′, prenatal maternal or fetal complications/treatments, fetal radiological signs, age at diagnosis, possible pathogenetic events, site of perforation, treatment of perforation, associated sepsis, and outcome in terms of mortality.

In addition, a systematic review of the literature was performed following PRISMA guidelines by two authors separately (CI and AB), searching the MEDLINE and SCOPUS electronic databases using the MeSH terms “neonate” and “gastric perforation.” In an attempt to include as many reported patients as possible, all full-text articles, including case reports and letters to the editor were retrieved and analyzed, provided they reported data on neonates with gastric perforation confirmed at surgery. Relevant studies were also searched in the reference lists of retrieved studies. We applied a date limit in order to include in the study only recent articles (published between 2005 and 2017) and written in English language. As a consequence, no conflicts had to be resolved. The variables extracted were the same as those for our retrospective series.

Statistical analysis was performed using Mann–Whitney test for continuous variables and Fisher’s exact test for categorical variables, results are reported as median (range) or prevalence, and *p* value < 0.05 was considered as statistically significant.

This study was approved by the Institutional Review Board from our institution (201502P003480).

## Results

Between January 2009 and December 2017, we treated eight consecutive patients for NGP. All were transferred from referring hospitals once the perforation was diagnosed. None had prenatal findings that suggested gastric perforation. In three cases prenatal ultrasound showed polyhydramnios. Three patients were preterm, two late preterm (37 weeks gestation) and three were born at term. The mothers of all preterm patients received steroids, 2 weeks before delivery in two (neonates born at 34 weeks gestation), and shortly before delivery in the third (neonate born at 32 weeks gestation). Mean BW was 2500 g (range 1,400–3200 g). The median age at presentation was 3 days of life; only one patient had a late onset (day 27) of gastric perforation. This case followed gap measurement for esophageal atresia (OA) using a Hegar dilator inserted through the gastrostomy to identify the length of the lower esophageal pouch (4). Four patients received mechanical ventilation support before gastric perforation occurred. Three patients had been fed before the perforation. The perforation was considered spontaneous in two cases (one detected shortly after birth) and secondary to trauma in the remaining six patients: five nasogastric tube decubitus and/or bag ventilation, one hegar dilator trauma. Associated malformations were OA with tracheo-esophageal fistula in two cases, duodenal web in one, and intestinal malrotation in one. All patients underwent open gastrorrhaphy, associated with a gastrostomy in three. Further management included correction of malrotation (Ladd’s procedure) and simple duodenotomy with duodenal web excision in one patient each. The infants with OA underwent closure of tracheo-esophageal fistula and direct esophageal anastomosis. Three patients developed a severe sepsis, treated with intravenous broad-spectrum antibiotics and antimycotic drugs. None of the patients of our series died.

**Figure 1:**
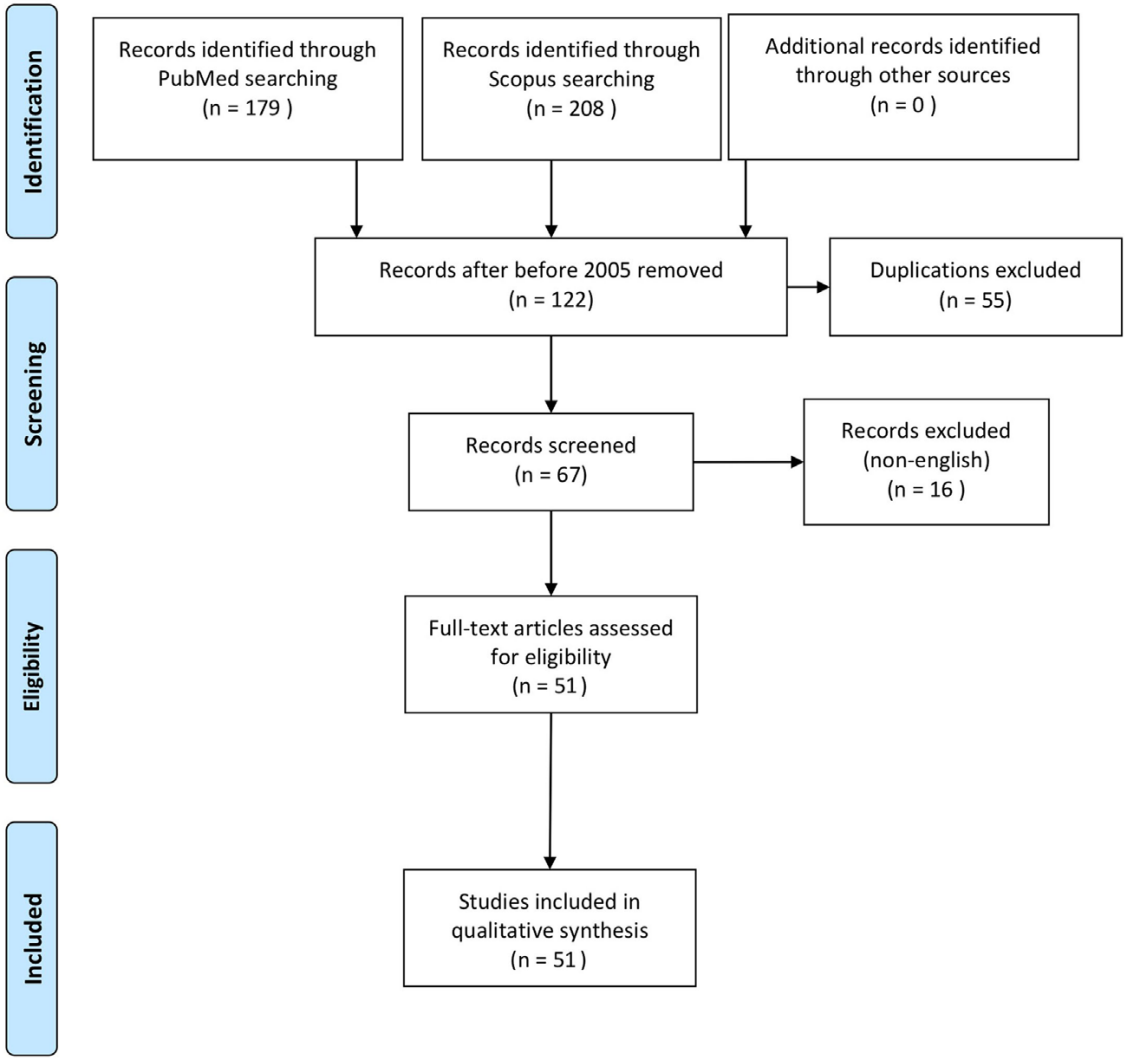
PRISMA 2009 flow diagram of systematic review of the literature (54).

The systematic review of the literature allowed to identify 51 articles (Figure 1) describing 200 further cases of NGP (1–3, 5–26, 30–52, 55–57). One patient was excluded from this study because managed conservatively and the perforation could not be confirmed, leaving 199 patients for the analysis (Table 1).

**Table 1:** Literature review.

Reference	Year	Pts (nr)	GA (weeks)	BW (kg)	Idiopathic	Secondary	Death	Sepsis (nr)
Rijhwano and Sunil (41)	2005	1	Unspecified	Unspecified	0	1	1	1
Hall and Ward (40)	2005	1	27	1.0	0	1	0	1
Abadir et al. (39)	2005	4	35 (29–39)	Unspecified	3	1	1	Unspecified
Im et al. (38)	2005	1	40	Unspecified	1	0	0	0
Kawase et al. (6)	2006	2	30	1.1	0	2	Unspecified	0
Gluer et al. (7)	2006	2	35 (32–38)	2.5	0	2	0	1
Woo et al. (21)	2006	1	31	1.9	1	0	0	0
Ebenezer et al (8)	2007	1	38	Unspecified	0	1	0	1
Korhonen et al. (9)	2007	1	37	3.1	0	1	0	1
Duran et al. (26)	2007	5	29 (28–30)	2.1 (0.6–3.6)	1	4	3	1
Esposito et al. (10)	2008	1	34	2.9	0	1	0	0
Lin et al (2)	2008	15	38 (32–38)	3.0 (2.0–3.6)	15	0	7	5
Asabe et al. (36)	2009	4	Unspecified	Unspecified	0	4	3	Unspecified
Anatol and Vilcov (37)	2009	2	37	Unspecified	0	2	0	Unspecified
Khan and Akhtar (12)	2010	1	37	1.8	1	0	0	0
Kshirsagar et al. (25)	2011	3	38 (33–38)	2.6 (2.1–3.1)	3	0	2	2
Lee et al. (52)	2011	1	33	2050	0	1	0	0
Kella et al. (50)	2011	14	38 (36–40)	2.3 (1.6–3.0)	6	5	3	3
Gasparella et al. (35)	2011	1	Unspecified	1.5	0	1	Unspecified	Unspecified
Rathod et al. (34)	2011	6	Unspecified	Unspecified	0	6	2	2
Arpit et al. (49)	2012	2	40	Unspecified	0	2	1	0
Joshi et al. (11)	2012	1	32	1.2	0	1	1	1
Khan et al. (13)	2012	1	36	1.6	0	1	0	1
Terui et al. (3)	2012	11	37 (33–40)	2.7 (1.5–3.2)	6	5	4	3
Ghribi et al. (14)	2013	7	35 (33–38)	1.6 (1.1–2.9)	1	6	6	3
Jactel et al. (15)	2013	8	36	2.3	5	3	3	5
Jiang et al. (16)	2013	1	38	2.8	0	1	0	0
Bos et al. (17)	2013	2	31 (26–37)	2.0 (1.0–3.0)	0	2	0	2
Mathur and Gupta (18)	2013	1	34	1.9	0	1	0	1
Okechukwu et al. (55)	2013	4	Unspecified	Unspecified	3	1	3	0
Erdogan (51)	2013	1	38	Unspecified	1	0	0	1
Ksia et al. (47)	2013	1	Unspecified	Unspecified	0	1	1	0
Lawther et al. (48)	2013	1	34	2.8	1	0	0	0
Byun et al. (23)	2013	9	38 (24–40)	2.9 (0.7–4.0)	7	2	2	2
Uettwiller et al. (46)	2014	1	36	Unspecified	0	1	0	0
Mahgoub et al. (19)	2014	1	24	0.7	0	1	1	1
Gupta et al. (22)	2014	1	Unspecified	Unspecified	1	0	0	1
Aydin et al. (1)	2015	1	30	Unspecified	1	0	1	0
Yang et al. (5)	2015	13	37 (28–39)	2.6 (1.3–3.5)	0	13	4	9
Abdullahi et al. (56)	2015	2	38 (38–38)	Unspecified	1	1	Unspecified	0
He (57)	2015	1	37	2.9	1	0	0	0
Mai et al. (33)	2015	1	33	Unspecified	0	1	0	0
Lee et al. (24)	2015	5	32 (28–34)	2.8 (1.2–3.0)	2	3	0	1
Raa et al. (20)	2016	1	35	1.9	0	1	0	0
Antabak et al. (42)	2016	1	40	3.4	0	1	0	Unspecified
Morsi et al. (31)	2016	1	Unspecified	Unspecified	Unspecified	Unspecified	Unspecified	Unspecified
Piplani et al. (43)	2017	1	Unspecified	Unspecified	0	1	Unspecified	Unspecified
Reyna-Sepulveda (44)	2017	1	27	Unspecified	1	0	0	1
Babayigit et al. (30)	2017	8	26	0.9	3	5	Unspecified	5
Tang et al. (32)	2017	1	40	2.9	0	1	0	0
Sato et al. (45)	2017	42	33^a^	2.8^a^	Unspecified	Unspecified	4	Unspecified

*Data are reported as single value when case report or median (range), when more than one case and data are available*.

*^a^Mean*.

*BW, birth weight; GA, gestational age; pts, patients*.

Prematurity was reported in 49% of patients. The pathogenesis was reported in 168 patients (including our series) and considered idiopathic in 79 (47%). The most frequent causes in non-idiopathic cases were: traumatic procedure (mechanical ventilation, esophageal intubation, NG tube insertion), ischemic insult, antenatal drugs (e.g., NSAID), or secondary to associated anomalies (Table 2).

**Table 2:** Causes of neonatal gastric perforation in non-idiopathic cases retrieved from the Literature and present series.

Etiology	Number of cases
Iatrogenic (e.g., MV, esophageal intubation, NG tube insertion…)	20
Necrotizing enterocolitis/sepsis	13
Duodenal/jejunal obstruction	11
Drugs	11
Esophageal atresia/tracheo-esophageal fistula	10
Diaphragmatic eventration/CDH	5
Midgut volvulus/intestinal malrotation	5
Pyloric atresia/stenosis	5
Abdominal wall defect	2
Lactobezoar	2
Bananas ingestion	2
Gastric hernia/volvolus	2
Zygomycosis	1

*CDH, congenital diaphragmatic hernia; MV, mechanical ventilation; NG, nasogastric*.

The most common site of perforation was the greater curvature (50%). Overall survival rate was 73%. Considering a total number of 207 patients (including our series), data on factors associated with mortality are available for 152 patients (Table 3). Survivors and non-survivors (NS) were similar in terms of prevalence of preterm birth, BW, Apgar score, prenatal manifestations, number of outborn patients, associated anomalies, and age at diagnosis. Only the prevalence of sepsis was statistically higher in NS (*p* < 0.0001).

**Table 3:** Clinical characteristics.

	Overall (166 pts)	*S* (103 pts)	NS (49 pts)	*p*
Prematurity (%)	45	43	50	0.6665
Birth weight (kg)	2.7 (0.6–4)	2.6 (0.7–4)	2.5 (0.6–3.6)	0.1921
1 min Apgar	7 (1–8)	8 (5–8)	8 (1–8)	0.6332
5 min Apgar	8 (4–9)	8 (8–9)	8 (4–9)	0.9859
Prenatal alterations (%)	21	19	24	0.7855
Outborn (%)	42	43	42	1.0000
Associated anomalies (%)	50	53	46	0.6468
Age at diagnosis (days)	3 (1–27)	3 (1–27)	3.5 (1–18)	0.8890
Sepsis (%)	36	24	61	0.0001

*Overall and comparison between survivors (S) and non-survivors (NS). Note that data on survival were available for 152 patients*.

## Discussion

Neonatal gastric perforation is an uncommon life-threatening entity in neonates, with several ill-defined aspects (58). This is the first study performing a systematic review of the recent literature to try to define pathogenetic and prognostic factors in neonates with gastric perforation.

In this study, we found that prematurity or low BW is frequently associated with the development of NGP. If cases with obvious anatomical predisposing factors (such as gastric outlet obstruction) or traumatic events are excluded, the etiology of NGP is still controversial, but prematurity, low birth weight (LBW), severe infections, and hypoxia are considered as contributing factors (3, 26). In this study, prematurity was reported in a substantial proportion on patients with NGP. Accordingly, in 2008 Lin and colleagues reported 15 cases and reviewed the literature, finding higher incidence of NGP among LBW babies (52%) in particular, in extremely LBW neonates, and suggested that premature infants are more prone to develop a spontaneous gastric perforation due to the immaturity of the gastric tissue (2). Similarly, in the survey from Sato et al. (45), 11 out of 42 neonates with gastric perforation were ELWB. Prematurity may predispose to NGP through several factors, including defects of the gastric muscle wall (especially in immature tissues of preterm babies), lack of intestinal pacemaker cells, and lack of C-KIT mast cell (27). Gastric smooth muscle cells (SMC) share with the interstitial cells of Cajal (ICC), the undifferentiated precursor stage. The expression of C-KIT, a tyrosine kinase receptor determines the development of ICC or SMC. The ICC plays an important role for the coordination of gastric motility, inducing slow rhythmic contractions of the gastrointestinal tract and neurotransmission. A condition of decreased ICC, as in premature infants, may determine a hypomobility of the gastrointestinal tract and predispose to spontaneous NGP (2). Accordingly, in 7 neonates with gastric perforation, Ohshiro et al. (53) found no ICC in the stomach of 3 patients and reduced ICC in the other 4, as compared to 10 control neonates. Incoordination and immaturity of esophagogastric motility, typical of premature neonates, are suggested as a possible mechanism causing increased intragastric pressure, thereby predisposing to gastric rupture (59). However, when gastric rupture occurs solely from overdistension, the rupture is usually along the lesser curvature (60). In present series, and collected patients from the systematic review of the literature, the perforation was most commonly in the greater curvature, suggesting that also other factors may play a role in gastric rupture in these patients. In premature infants, the gastric wall may have an intrinsic fragility. As a consequence, relatively minor traumatic events (such as the nasogastric tube decubitus and/or bag mask ventilation, hard crying or neonatal swallowing incoordination) or abnormally high intragastric pressure may bring about a local gastric wall disruption and perforation. In addition, in the gastrointestinal system of the premature infant, circulatory regulation may not be fully developed, possibly causing a reduced blood flow during hypoxic events or non-steroid anti-inflammatory drugs (NSAID) treatments. It is possible that the immature vascular bed may participate in the development of NGP, as supported by the association of NGP with necrotizing enterocolitis or NSAID administration (6, 61). Finally, neonates, particularly preterm ones, may present increased susceptibility to infectious complications, both bacterial and fungal, that have been associated with the development of gastric perforation (9, 18).

Anatomical anomalies leading to gastric outlet obstruction may predispose to NGP both in term and preterm neonates. One of our patients who developed NGP had associated duodenal web. He was also preterm (34 weeks gestation) and it is not possible to define without uncertainty whether the prematurity was the major pathogenetic element or if the interplay between gastric outlet obstruction and prematurity contributed to the perforation. However, the gastric outlet obstruction may have played a role in the development of NGP through an abnormal increase of intragastric pressure. In 2008, Esposito and colleagues reported a case of antenatal gastric perforation associated with Bochdaleck diaphragmatic hernia, presenting at birth as pneumothorax and perforative peritonitis (10). Other extremely rare condition, such as Carmi Syndrome can be associated with NGP. The presence of congenital pyloric atresia associated with epidermolysis bullosa, may predispose to high intragastric pressure, representing a risk factor for NGP (11).

Clinical presentation of NGP is usually characterized by acute abdominal distention often associated with sepsis and respiratory failure. Although up to 70% mortality rate has been reported in NGP, the advance of the technology for respiratory and hemodynamic support improved the survival rate of patients with NGP during past years (27). In our study, sepsis was the only variable significantly associated with mortality. Therefore, in a neonate with suspected NGP, signs of hypovolemic or septic shock must be actively checked, as timely treatment of this complication appears to be the single most important factor influencing survival. Although conservative management of neonatal pneumoperitoneum with a peritoneal drain has been proposed, especially in critically unstable patients, when the patient is stabilized, surgery is the ideal treatment in this uncommon disorder, also to contribute to the management of septic shock (5, 28, 29). Surgical treatment may be limited to necrotic tissue excision and gastrorrhaphy, if the abdominal cavity has only limited or no flogistic changes. In case of important inflammation, an associated gastrostomy should be considered. Rarely partial gastrectomy was performed in case of massive gastric disruption. In addition, the underlying anatomical cause must be recognized and treated, if present.

This study has some limitations. First, the rarity of the condition itself. To try to overcome this limitation and to increase the study population, we have performed a systematic review of the literature. We have empirically limited to 2005, the date of the review to include only the series/cases treated more recently, to avoid potential biases related to treatment changes on mortality rate. The inclusion of all types of studies, including case reports and editorials provided they described patients, without preclusions related to the country of origin, may be a source of heterogeneity, and introduce some biases related to treatment availabilities. However, the fact that prematurity or low BW was not associated with the outcome in terms of mortality suggests that studies were not that heterogeneous in terms of treatment availabilities.

## Conclusion

In conclusion, gastric perforation is an uncommon, but life-threatening condition of the neonates. In present series and systematic review of the literature, the only factor affecting survival was the development of sepsis, probably related to prompt diagnosis and surgical treatment and high quality of pre- and post-operative care. In the past 10 years, the overall survival rate has drastically improved, from less than 25% to over 50%, probably thanks to the progress in neonatal intensive care procedures.

## Ethics Statement

This study was approved by the Institutional Review Board from our institution (201502P003480).

## Author Contributions

FM, FF, and CI conceived and coordinated the study. CI and AB collected data. CI and FM analyzed data and wrote the paper. FM and PB reviewed the results and approved the final version of the manuscript.

## Conflict of Interest Statement

The authors declare that the research was conducted in the absence of any commercial or financial relationships that could be construed as a potential conflict of interest.
